# On the Importance of Early Blood-Letting in Acute Organic Affections

**Published:** 1808-12

**Authors:** 


### *On the Importance of early Blood-letting in Acute Organic Affections.* By Dr. Adams.

OUR last number contains a very useful communication from Dr. Kinglake on the Use and Abuse of Blood-letting. It is not my intention to notice the objections which will be made by some to the manner in which the buffy incrustation is formed under certain circumstances. It is enough that the author describes the result of his own experiments, by which every one is left to assign the less obvious causes. But as he has shown with much practical accuracy, the disadvantages of too free blood-letting, it may not be amiss to remark, that there are some cases in which the early and free use of the lancet is the only means of preserving our patient. This is the more necessary, because it not unfrequently happens, that after a copious, but too long deferred blood-letting, the patient shall die with all the symptoms of debility, from a too free use of the only remedy which could have saved him.

Master ———, a somewhat delicate child, about 10 years old, was seized, during the present autumn, with violent cough and pain in the abdomen. The gentleman who attended him, describes his pulse quick, and face suffused with a dark red colour. On the following day the symptoms remained the same. On the next, the febrile symptoms toms increased towards the night. On the 4th, [of the disease] the symptoms were greatly increased, and I was consulted about three in the afternoon. At this time, the colour of the patient's cheeks was purple; his respiration more like panting than breathing; his pain considerable about the region of the heart. On placing my hand over the left breast, the efforts made by the heart to perform its necessary functions, produced a sensation which cannot be described. There seemed two or three pulsations, one much more violent than the rest, and almost all at the same instant. The pulse at the wrist was feeble and irregular, so that it was scarcely possible to count it.

I directed a vein to be instantly opened by a large orifice, and staid to determine the extent to which the bleeding should be carried. After drawing about six ounces of blood, the operator could procure no more, though much pains were taken; and the patient remaining recumbent, did not faint. The symptoms were, however, all moderated, and the pulse more regular. After prescribing purgative and other remedies, directions were given to the surgeon to repeat the bleeding the moment any severe symptoms returned, to be governed by the effect in the quantity taken away, or if he should be doubtful of keeping the vein open as long as the pain continued, that I would be careful to be readily found, should he wish to see me.

On the following morning, I was informed that our patient had continued, during the evening, without any considerable paroxysm, though his breathing and pulse had not recovered themselves; but early in the morning, the pain having returned, the bleeding was repeated to the quantity of ten ounces.

The blood taken the first time had no particular appearance, but this might have arisen from the slow manner in which it trickled down the arm and along the basin. That taken afterwards, escaped by a full stream, coagulated freely, and exhibited a buffy appearance. The purgative had operated, and the patient was freer from pain. On being introduced to him, however, this relief appeared partly the effect of a state approaching to syncope. His cheeks were of a much darker purple, his respiration as before, and the pulsation at the heart, though less violent, was equally irregular; at the wrist it was obscure. Early in the afternoon my visit was repeated. His violent pain had not returned, but all the other symptoms remained; and in addition to these, his face was cold, and suffused with moisture. At half past six he died. Leave was obtained to open the

 body, body, which was done by Mr. Taunton, and the following appearances noticed by him.

"The thymus gland more evident than is usual at that age.

Many lymphatic glands greatly enlarged between the trachea and superior cava, by which the latter was pushed more forward, and a little to the right side.

The surface of the lungs inflamed, and the lobes adhered at the fissures.

The substance of each lung more firm than is usual in health, being barely boyant in water, contained tubercles, and in many parts pus.

The pericardium contained more water than usual, perfectly transparent, and yellowish. The coronary vessels and vasa vasorum on the pulmonary artery and aorta, were very turgid, so as to indicate a great determination of the blood to those parts.

The right auricle contained a large yellow coagulum, almost filling the cavity, adhering to it, and containing blood-vessels, which communicated with the substance of the auricle. This coagulum was continued into the ventricle in an oblong form. To this cavity it was more firmly attached by communicating vessels. There was also a considerable membrane of effused lymph adhering to the inner surface of the right ventricle.

Some points of apparently incipient ossification were found on the mitral valves.

The liver rather large, but the abdominal viscera were healthy, except that the mysenteric glands were enlarged, and some of them hardened."

The above description sufficiently shows the cause of death in this patient, and proves to the most sceptical conviction, that nothing but very early and copious bleeding could have prevented those effects of inflammation, which must have taken place in some of the first paroxysms. Yet, if the body had not been opened, suspicion might have arisen that the free bleeding produced the subsequent debility and death.

It is stated that ossification seemed to have begun in the valvulæ mitrales. In conversing with the friends of the deceased, I was informed that about twelve months ago he had symptoms somewhat similar to his last illness, tho' much milder; that he was bled and recovered, but from that time it had been remarked, that particularly after eating his colour was always very high, changed frequently, and his cheeks sometimes of a purple hue. He was also also never fit for the exertions of boys of his own age. It is too much to suspect that the beginning ossification in the valvulæ mitrales was the effect of inflammation during that previous illness? It is, I believe, an observation of Mr. Hunter's, that certain parts become bony, in order to support themselves under particular circumstances. This is usually, perhaps, a slow progress; but is there any thing inconsistent with pathology, in supposing that such a provision being made, may take place under any circumstances of action more powerful than parts, by their original structure can support? That a deposition of earthy matter in the heart may be the effect of inflammation, appears tolerably ascertained by the following case, which I had occasion to describe immediately after the event.

A boy, about 14 years of age, died in St. Bartholomew's Hospital, after three days of intense pain about the region of the heart, which yielded to no remedies, and during which, a watery effusion suddenly appeared in one foot, and was as suddenly transferred to the scrotum.

The late Mr. Sheldon opened the body, and a deposition of earthy matter was found in the substance of the heart.—I was present at the opening of an older subject, by Mr. Hunter; I did not see the patient during life, but his attendants described his pain as sufficiently intense to become the cause of his death. A deposition of earthy matter was found in the inside of the aorta, the surface of which, under the deposition, was rough.

If these two instances are admitted to be the effect of inflammation, may not such a degree as the patient can survive, prove the beginning of some kinds of aneurism, and thus terminate by a more tedious death?

It is probable that such cases are not very common. Dr. Baillie, whose opportunities have been larger than most men's now living, informs us that he has never seen this earthy deposition. It may seem strange, that one of so much more confined opportunities should have met with two instances. To this I can only say, that the bodies were both opened, on account of the peculiarity of the symptoms; and that the parts suspected, were particularly examined. The first circumstance shows the rariety of such incidents; and it may be added, that had either of these bodies been brought into a dissecting-room, the parts might have been preserved for injection, in which case the disease would not have been discovered.

These remarks, however, are chiefly intended to direct practitioners to one most important object; namely, in all case cases of death after violent pain in the region of any important organ, to ascertain as often as possible, whether relief might not have been obtained by early depletion; or if the patient has died after the full use of that remedy, whether it would have been possible to have saved him by any other means.

If the instructions of Celsus are just, to bleed, *ubi quisquis intolerabilis dolor est*, it requires no argument to show the absurdity of resting till the object for which we begin is accomplished.—But the question cannot be fairly determined without frequent examinations after death.

*Bridge Street.*

